# 靶向肽在递送siRNA进行肺癌治疗研究中的应用

**DOI:** 10.3779/j.issn.1009-3419.2014.09.06

**Published:** 2014-09-20

**Authors:** 红林 高, 鉴峰 刘, 娜玲 宋

**Affiliations:** 300192 天津，中国医学科学院北京协和医学院放射医学研究所，天津市放射医学与分子核医学重点实验室 Department of Tianjin Key Laboratory of Radiation Medicine and Molecular Nuclear Medicine, Institute of Radiation Medicine, Peking Union Medical College, Chinese Academy of Medical Sciences, Tianjin 300192, China

**Keywords:** 肺肿瘤, 靶向肽, siRNA, Lung neoplasms, Target peptide, siRNA

## Abstract

肺癌被认为是全球发病率最高的一种恶性肿瘤，其对化疗药物不敏感且易产生耐药性，因此增强肺癌药物治疗效果是近年来研究的热点。siRNA是一种小RNA分子，可以沉默与之互补的目标mRNA，是一种基因治疗手段。靶向肽是一类小分子多肽，它可以与siRNA联合使用，利用其与肿瘤表面物质特异性结合发挥靶向作用。联合靶向肽的特异性和siRNA的治疗作用，增加siRNA在靶点位置的聚集，增强沉默效果，可以提高肺癌对药物的敏感性并降低耐药作用，进而增强肺癌治疗效果，为肺癌的靶向治疗提供新的方向和策略。本文将对靶向肽在递送siRNA进行肺癌治疗研究中的应用作一简要综述。

## Abstract (en)

Lung cancer is considered a kind of malignant tumors of the world highest incidence. As it is not sensitive to chemotherapy and easy to produce drug resistance, improving effect of anticancer drug becomes a research focus recent years. siRNA, small interfering RNA, can silence complemenary mRNA which is a kind of gene therapy. Target peptides are small molecular peptides which specifcally bind to tumor surface materials. When used with siRNA, target peptides can increase accumulation of siRNA in tumor cells and enhance the silencing effect. As result, drug resistance of lung cancer reduced and the effect of therapy can be improved. Tis method provides new direction and strategy for targeted therapy of lung cancer. Tis article will make a brief overview of target peptides applying in siRNA dilivery for the research of lung cancer treatment.

随着城市环境及人们生活规律的改变，我国肺癌发病率呈逐年上升趋势。肺癌因其恶性程度高，疗效差，不易早期诊断等被认为是所有癌症中的头号杀手，它可分为小细胞肺癌(small cell lung cancer, SCLC)和非小细胞肺癌(non-small cell lung cancer, NSCLC)两大类，其中后者大约占85%^[1]^。大部分的肺癌患者不易早期发现，在晚期确诊时常规的化疗效果不明显，化疗药物的毒副作用及患者产生耐药性往往是肺癌治疗失败的主要原因。近些年兴起小干扰R NA(small interfering R NA, siR NA)的靶向递送可以提高肿瘤对药物的敏感性降低耐药作用，增强治疗效果，某些siR NA单独投放便可抑制肿瘤的生长及迁移^[2]^。靶向肽因其具有与肿瘤表面受体特异性结合的功能，若将靶向肽siRNA联合应用，将明显提高siRNA治疗效果，这也成为越来越多研究者关注的热点^[3]^。本文重点总结了靶向肽在递送siR NA进行肺癌治疗研究中的应用。

## 1 siRNA及靶向肽概述

### 1.1 siRNA

siRNA是含有5个-21个核苷酸的小RNA分子，在RNA干涉(RNA interfering, RNAi)中起中心作用，对特定mRNA进行降解。RNAi的原理是利用具有同源性的双链RNA诱导序列特异的目标基因的沉默，迅速阻断基因活性。根据RNAi原理，利用短的双链RNA沉默与其同源的特异基因，这种基因沉默的方法已成为抗癌治疗的新趋势。这些短的双链RNA有几种类型，包括siRNA、microRNA(miRNA)、小的非编码RNA (tiny non-coding RNA, tncRNA)和短发卡RNA(short hairpin RNA, shRNA)，其中siRNA运用最为广泛。利用siRNA基因沉默进行抗癌治疗的研究在近些年兴起，例如Yang ^[4]^运用阳离子脂质体来递送血管内皮生长因子(vascular endothelial growth factor, VEGF) siRNA以达到抗脑胶质瘤的效果，Bagheri^[5]^通过siRNA介导的DNA断裂因子45(DNA fragmentation factor 45, DFF45)沉默来增强阿霉素抗乳腺癌细胞的效果，Pietschke^[6]^研究表明siRNA可以作为治疗前列腺癌的有效方法，Shi^[7]^利用siRNA沉默人端粒酶逆转录酶(human telomerase reverse transcriptase, hTERT)在体内外均抑制宫颈癌细胞的生长和迁移。类似的siRNA研究还有很多，其限制性在于缺乏有效的递送载体将核酸投递给肿瘤细胞^[8]^，鉴于这一点，越来越多的递送载体被发现和研究，其中靶向肽因其良好的靶向作用逐渐被广泛研究。

### 1.2 靶向肽

靶向肽(target peptide)是特异性结合肿瘤表面标志物的一类小分子多肽，包含的氨基酸数量一般少于50个^[9]^。1985年，Smith^[10]^第一次将外源基因插入丝状噬菌体f1的基因Ⅲ，使目的基因编码的多肽以融合蛋白的形式展示在噬菌体表面，从而创建了噬菌体展示技术，自此开启了靶向肽研究的新篇章。获取靶向肽的方式有多种，可以改进已知多肽，构建多肽受体的分子结构，或者搜索结合肽文库，结合肽文库包括噬菌体展示文库、细菌展示文库、一珠一化(one-bead one-compound, OBOC)和定位扫描合成肽库(Positional Scanning-Synthetic Peptide Combinatorial Libraries, PSSPCLs) ^[3]^。靶向肽有很多优点，它在纳摩尔浓度就可显露对靶标的高特异性，并且本身低毒，对肿瘤周边的正常组织无明显损害作用，在非靶点位置能快速被清除，同时它容易合成并优化以更好结合靶标，也可以调整结构增加对蛋白酶降解的稳定性、延长在血液循环中的半衰期和增强毛细血管通透性^[9]^。目前靶向肽已广泛运用于肿瘤影像和治疗的研究中，本文着重关注其在递送siRNA抗肺癌的研究。

## 2 靶向肽在递送siRNA进行肺癌治疗研究中的应用

肺癌的发生及发展需要经历一系列复杂的过程，多信号传导机制参与其中，有些多肽可以抑制某些蛋白过基因表达来阻断某一信号通路而发挥抑癌作用，这类多肽同时可以作为靶向肽在递送siRNA过程中发挥靶向作用。此外，肺癌细胞表面存在许多类细胞表面分子，可以作为多肽的靶点，一些多肽利用这些靶点发挥靶向作用。有些多肽自身即可作为递送siRNA的载体并发挥靶向作用。本文从靶向肽发挥靶向作用及其作为载体在递送siRNA两方面来综述siRNA递送治疗肺癌的新进展。

### 2.1 靶向肽作为靶向剂递送siRNA

#### 2.1.1 靶向肽拮抗剂G

拮抗剂G(antagonist G)是神经递质P物质6-11序列的六肽类似物，通过人工合成，其序列是RWFWLM，其中W均为D-构型，功能是阻断神经肽生长因子。SCLC是转移性神经内分泌肿瘤，受自分泌和旁分泌的调节，拮抗剂G可以阻断神经肽生长因子结合到肿瘤细胞受体上而发挥抑癌作用，研究^[11]^表明它通过生成过氧化物诱导SCLC的凋亡。此多肽曾与包裹阿霉素的脂质体融合实现阿霉素对SCLC的靶向抑制作用^[12]^。Santos等^[8]^设计脂质体包裹siRNA后再与拮抗剂G共价结合形成稳定的颗粒，输送到SCLC中发挥作用，这个系统可以作为日后输送siRNA治疗SCLC的参考。

#### 2.1.2 靶向肽Disruptin

表皮生长因子受体(epidermal growth factor receptor, *EGFR*)发生突变是肺癌发生的一种方式，抑制其表达及活化可以达到抑制肺癌生长发展的效果。EGFR通过配体诱导形成二聚体而活化，激活下游信号传导途径。Ahsan等^[13]^新合成一种称作Disruptin的19个氨基酸多肽，结构中包含EGFR的SVDNPHVC部分，可以阻断热休克蛋白90结合EGFR，并且抑制EGFR二聚化从而引起其降解，这一多肽引起*EGFR*突变阳性的肿瘤细胞如UMSCC1和肺癌NCI-H1975细胞表面EGFR降解，因此癌细胞生长受到抑制。

#### 2.1.3 cRGD

整合素过表达在许多癌症如肺癌细胞表面，可以作为研究的靶点。cRGD(cycle Arg-Gly-Asp)是从噬菌体展示库筛选得到可特异性结合整合素的靶向肽，cRGD在递送siRNA至肺癌细胞过程中发挥靶向作用那个在许多研究中体现。在Khatri等^[14]^的实验中，siRNA可渗透通过脂质体双分子层，与脂质体内部的钙磷酸盐络合在一起，包裹siRNA的脂质体再与cRGD结合以获得对A549肺癌细胞的靶向性。研究结果发现，连接cRGD的siRNA脂质体的A549细胞活力明显比单纯siRNA脂质体高，且细胞荧光强度高，表明肺癌细胞对具有靶向肽的siRNA吸收多。

如果递送的siRNA针对肿瘤生长及迁移的功能基因mRNA，其发挥的抑癌作用也会更大。Khatri^[15]^设计的siRNA纳米结构和cRGD接合物应用于NSCLC治疗中能够明显提高肺癌对吉他西滨盐酸盐的敏感性。该siRNA作用核糖核苷酸还原酶亚单位1(ribonucleotide reductase M1, RRM1) mRNA，*RRM1*基因编码RRM1的调节亚基，该酶是生物体内唯一催化4种核糖核苷酸还原生成相应的脱氧核糖核苷酸的酶，对细胞的增殖和分化起着调控作用。cRGD靶向NSCLC细胞表面的ανβ3整合素，RRM1 siRNA进入细胞内抑制RRM1的表达，肺癌细胞的生长活力降低，对吉西他滨盐酸盐的半数抑制浓度(50% inhibitory concentraton, IC_50_)值明显降低，表明siRNA增强肺癌细胞对药物的敏感性。

#### 2.1.4 YSA

EphA2是一个跨膜酪氨酸激酶受体，属于酪氨酸激酶受体(recetptor tyrosine kinases, RTKs)超家族成员之一，研究^[16]^发现其在肺癌细胞表面高表达。Ishikawa等^[17]^运用实时定量PCR技术发现有良好病症的患者病理Ⅰ期NSCLC表面高表达EphA2。12氨基酸多肽(YSAYPDSVPMMS or YSA)通过噬菌体展示技术获得，靶向肿瘤细胞表面EphA2受体^[18]^。Dickerson等^[19]^将包裹针对EGFR基因的siRNA的纳米颗粒通过N, N-双丙烯酰胺与YSA连在一起制成结合物，然后将结合物分别添加到EphA2阴性的Hey和EphA2阳性的SK-OV-3细胞中，其中YSA介导EGFR siRNA内吞进肺癌细胞。免疫印迹法确认两者EGFR量的变化，其后给予多西他赛，比较肿瘤对药物的敏感性。研究表明，经过siRNA的靶向递送，与Hey相比，Eph2阳性的SK-OV-3细胞EGFR表达明显降低，且癌细胞对药物的敏感性明显增强，正常组织损伤较小。

### 2.2 靶向肽自身作为载体递送siRNA

脂质体和纳米颗粒等作为siRNA的递送载体在抗癌研究中广泛应用，但是材料的生物相容性是基础研究走上临床必须要考虑的因素，不断有研究者发现了生物相容性好的新的多肽载体，不仅可以发挥其靶向性，还可以自身作为载体递送siRNA，使得siRNA在肿瘤靶点聚集，增强抗癌效果。细胞穿膜肽(cell-penetrating peptides, CPPs)作为一类新型的递送药物分子和siRNA等的载体，目前已有很多中CPP被设计出并应用于研究中。

#### 2.2.1 C6

Jafari等^[20]^介绍了一种18聚体的两亲氨基酸配对肽C6作为siRNA的递送载体，研究表明与脂质体2000相比，C6具有低毒性和细胞内吸收siRNA的高效性。C6是人工设计合成的CPP，带正电，通过与肿瘤细胞膜负电部分结合而透过细胞膜。C6M1是对C6的优化，其溶解度、二级结构和细胞内吸收等特性均有明显改善，使之成为更好递送siRNA的载体^[21]^。在Jafari^[22]^实验中，C6M1与siRNA在一定比例下形成稳定的复合物，表现出对血清RNase的稳定性，相比于裸RNA，该复合物在肿瘤细胞内siRNA的吸收也明显增加。

#### 2.2.2 hCT分支衍生物

Hoyer^[23]^设计合成了截短型人降钙素(human calcitonin, hCT)的分支衍生物，将其与siRNA非共价结合形成稳定复合物，该siRNA靶向人神经肽Y1受体(human NPY Y1 receptor, NPY1R)，其中NPY1R是G蛋白偶联受体(G protein-coupled receptors, GPCR)家族成员之一。研究过程中这个多肽不仅可以作为siRNA的载体，也可以与NPY1R特异性结合，使siRNA靶向肿瘤位点，发挥更强的沉默作用，实时定量PCR结果表明，相对于对照组，实验组NPY1R mRNA降低两倍多。

#### 2.2.3 TAT-A1

TAT是第一个发现的穿膜肽，通过噬菌体展示技术获得，被广泛研究和应用在核酸递送研究中。TAT-A1是中国农业大学Fang^[24]^新设计出的具有肿瘤靶向且可以递送siRNA的CPP，其中A1是特异性结合血管内皮生长因子受体1(vascular endothelial growth factor receptor-1, VEGFR1)的六肽，VEGFR1过表达在肿瘤如肺癌细胞表面，研究结果显示，VEGFR1过表达的肿瘤细胞内，TAT-A1复合物siRNA的细胞吸收和肿瘤位点聚集能力均优于TAT，因此可以作为siRNA治疗肺癌的一种方法。

#### 2.2.4 PR39

抗菌肽(antimicrobial peptides, AMPs)是一种小分子多肽，有些对肿瘤有抑制作用。PR39猪抗菌肽从细菌展示库中获得，作为一种穿膜肽，可以通过结合肿瘤细胞的负电部分而透过细胞膜。Tian等^[25]^将PR39作为针对STAT3的siRNA的载体递送siRNA，相对于裸siRNA，此系统能明显抑制乳腺癌细胞的侵入和迁移，其中PR39与siRNA的比例为90:1时STAT基因沉默效果最大。在Kim等^[26]^的研究中，能特异性结合STAT3的多肽APT可以通过阻断STAT信号通路而抑制肺癌细胞生长及迁移，其中APT由噬菌体展示技术获得，因此抗菌肽PR39递送STAT3 siRNA的设计未来可以有效应用于肺癌治疗研究。

## 3 结语

利用siRNA沉默目的基因的方式治疗肺癌是近年来研究的热点，siRNA自身可以发挥明显的抑癌作用，也可以增加肺癌对抗癌药物的敏感性，减少耐药发生，但因其在体内极不稳定，缺乏有效地递送系统和靶向性，单纯siRNA治疗的效率通常不高。靶向肽在递送siRNA的应用中可以显著增加siRNA在肿瘤靶点的集聚能力，使其更好地发挥其作用，尽管靶向肽应用于肺癌治疗研究具有广阔的应用前景，但目前尚存在许多问题待解决，包括如何获得肺癌发生及转移相关靶基因的siRNA；如何筛选出肺癌细胞特异性更好的靶向肽以及如何提高靶向效率；siRNA如何包裹在载体中，在血液循环过程中保持稳定，而进入肿瘤细胞后可以迅速释放；如何选择合适的载体使其在体内不易被排出且对正常组织无损害等。为解决这些问题，我们应该深入研究肺癌发生及发展的相关机制，筛选并设计出肺癌特异性高和容易实施的多肽，寻找安全无毒且易操作的载体，更好地实现与siRNA和靶向肽结合，最终使肺癌患者能够受益。
